# Hierarchical clock-scale hand-drawn mapping as a simple method for bronchoscopic navigation in peripheral pulmonary nodule

**DOI:** 10.1186/s12931-022-02160-0

**Published:** 2022-09-14

**Authors:** Chang-Hao Zhong, Zhu-Quan Su, Wei-Zhan Luo, Wan-Yuan Rao, Jia-Xin Feng, Chun-Li Tang, Yu Chen, Xiao-Bo Chen, Ming-Yue Fan, Shi-Yue Li

**Affiliations:** State Key Laboratory of Respiratory Disease, National Clinical Research Center for Respiratory Disease, Guangzhou Institute of Respiratory Health, The First Affiliated Hospital of Guangzhou Medical University, 151 Yanjiang Road, Guangzhou, 510120 China

**Keywords:** Bronchoscopy, Peripheral pulmonary nodule, Hand-drawn bronchoscopic navigation, Virtual bronchoscopic navigation, Diagnostic yield

## Abstract

**Background:**

A feasible and economical bronchoscopic navigation method in guiding peripheral pulmonary nodule biopsy is lacking.

**Objective:**

To investigate the utility of hierarchical clock-scale hand-drawn mapping for bronchoscopic navigation in peripheral pulmonary nodules.

**Methods:**

We developed a hierarchical clock-scale hand-drawn mapping for bronchoscopic navigation in peripheral pulmonary nodules. Patients with peripheral pulmonary nodules were recruited and assigned to two groups in this retrospective study, subjects in VBN group received conventional bronchoscopy in conjunction with virtual bronchoscopic navigation (VBN) and radial probe endobronchial ultrasound (RP-EBUS) for biopsy (VBN group), while HBN group underwent ultrathin bronchoscopy and RP-EBUS under the guidance of hand-drawn bronchoscopic navigation (HBN). The demographic characteristics, procedural time, operating cost and diagnostic yield were compared between these two groups.

**Results:**

Forty-eight patients with peripheral pulmonary nodule were enrolled in HBN group, while 42 in VBN group. There were no significant differences between VBN and HBN groups in terms of age, gender, lesion size, location and radiographic type. The time of planning pathway (1.32 *vs.* 9.79 min, *P* < 0.001) and total operation (23.63 *vs.* 28.02 min, *P* = 0.002), as well as operating cost (758.31 ± 125.21 *vs.*1327.70 ± 116.25 USD, *P* < 0.001) were markedly less in HBN group, compared with those in VBN group. The pathological diagnostic efficiency of benign and malignant disease in HBN group appeared similar with those in VBN group, irrespective of the size of pulmonary lesion (larger or smaller than 20 mm). The total diagnostic yield of HBN had no marked difference from that of VBN (75.00% *vs.* 61.90%, *P* = 0.25).

**Conclusions:**

Hierarchical clock-scale hand-drawn mapping for bronchoscopic navigation could serve as a feasible and economical method for guiding peripheral pulmonary nodule biopsy, providing a comparable diagnostic yield in comparison with virtual bronchoscopic navigation.

**Supplementary Information:**

The online version contains supplementary material available at 10.1186/s12931-022-02160-0.

## Background

Lung cancer remains the major cause of cancer related deaths worldwide [1]. Emerging evidence suggests that low-dose computer tomography (LDCT) for lung cancer screening is conducive to the early detection of solitary pulmonary lesions and improve the prognosis of the lung malignancies [2, 3]. However, over 70% of the suspected pulmonary tumor lesions develop as peripheral pulmonary nodule [4], presenting a particular challenge for tissue acquisition for further pathological evaluation.

Several bronchoscopic guidance modalities have been developed to improve the diagnostic yield of conventional bronchoscopy [5–8], including radial probe endobronchial ultrasound (RP-EBUS), virtual bronchoscopic navigation (VBN) and electromagnetic navigation bronchoscopy (ENB). VBN emerges as a navigation modality that employs chest high-resolution computed tomography (HRCT) imaging data to provide three-dimensional virtual images of bronchial route to the target peripheral pulmonary nodule [9]. Moreover, ultrathin bronchoscope, with an outer diameter ≤ 3.5 mm, has been recently implemented to access more distal airways with a good maneuverability for pulmonary lesion detection [7, 10].

An increasing number of patients with suspected lung cancer require tissue biopsy for pathological diagnosis. However, the majority of public hospitals in China have a challenge to perform accurate diagnosis of peripheral lung lesion, which might be in part due to the absence of bronchoscopic navigation system for financial limitation and a lack of sophisticated operator [5, 6]. Referring to Prof. Noriaki Kurimoto’s bronchial branch tracing method [11], the current study have introduced a hierarchical clock-scale hand-drawn mapping for bronchoscopic navigation based on HRCT image in conjunction with ultrathin bronchoscope, and employed VBN procedure for comparison, we hypothesized that the hand-drawn mapping for bronchoscopic navigation (HBN) could serve as a non-inferiority method for guiding peripheral pulmonary lesion biopsy compared with VBN system.

## Methods

### Bronchoscopic navigation mapping for lung biopsy

**Fig. 1:**
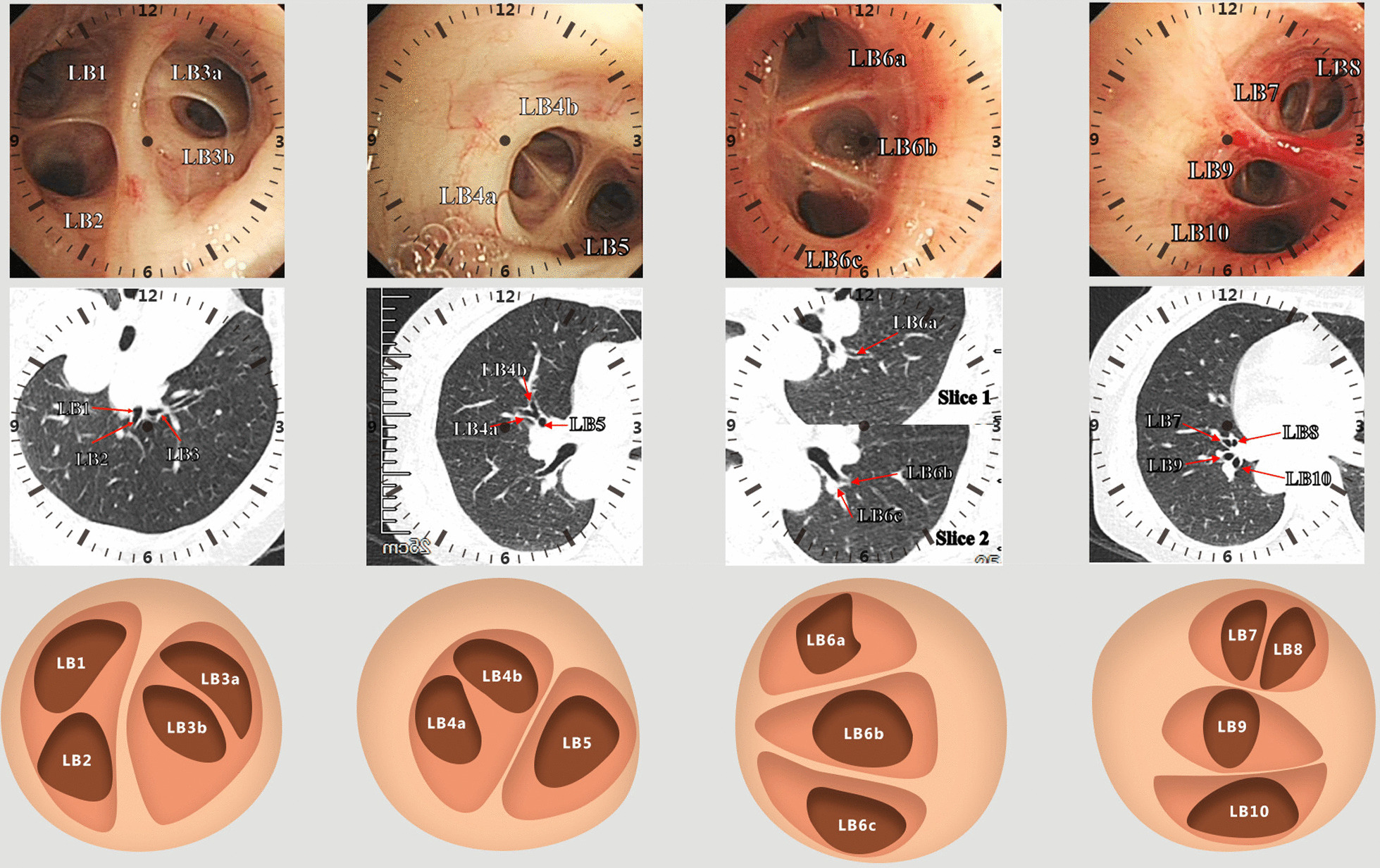
Bronchoscopy, CT image and navigation mapping of the right lung

**Fig. 2:**
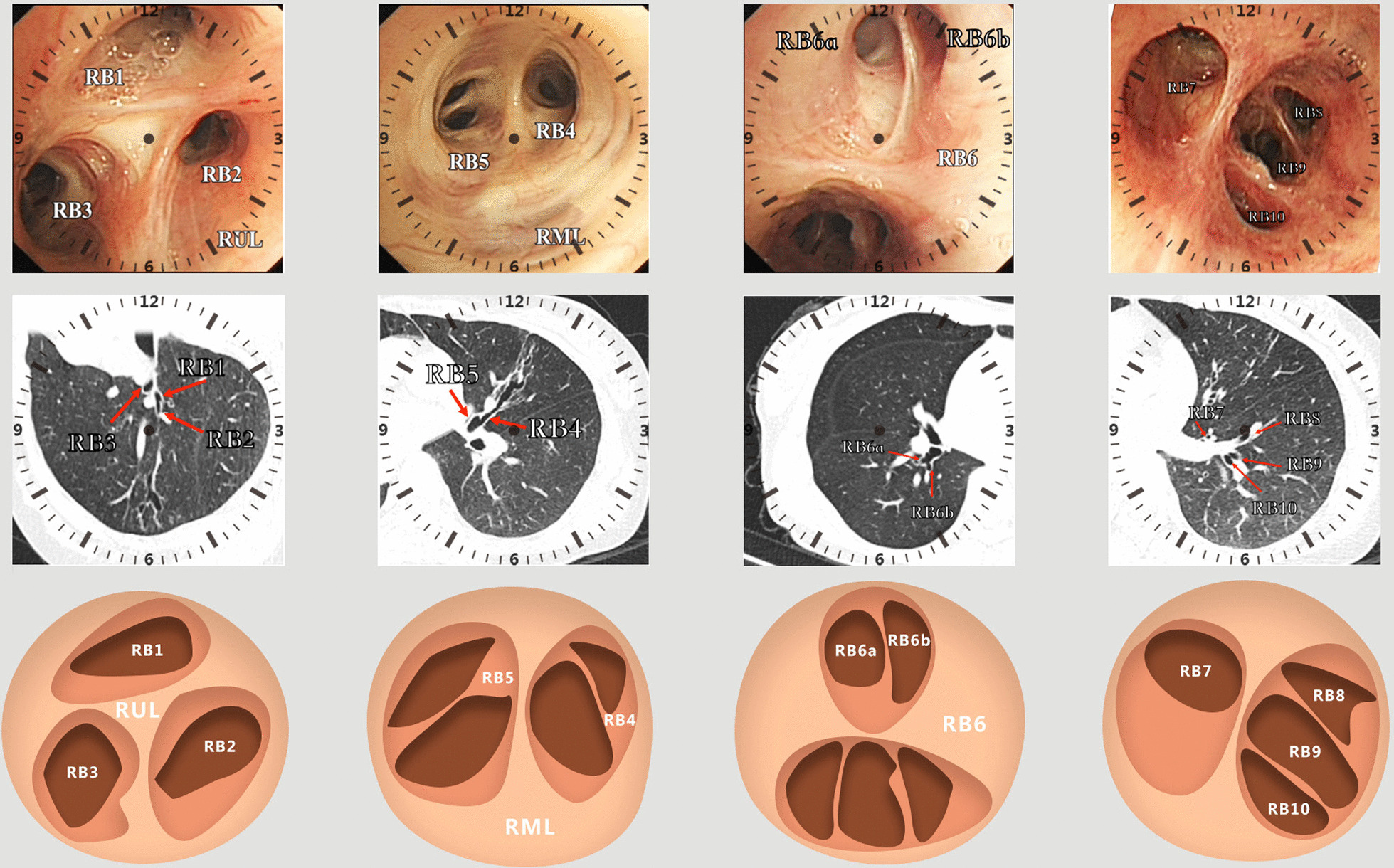
Bronchoscopy, CT image and navigation mapping of the left lung

**Fig. 3:**
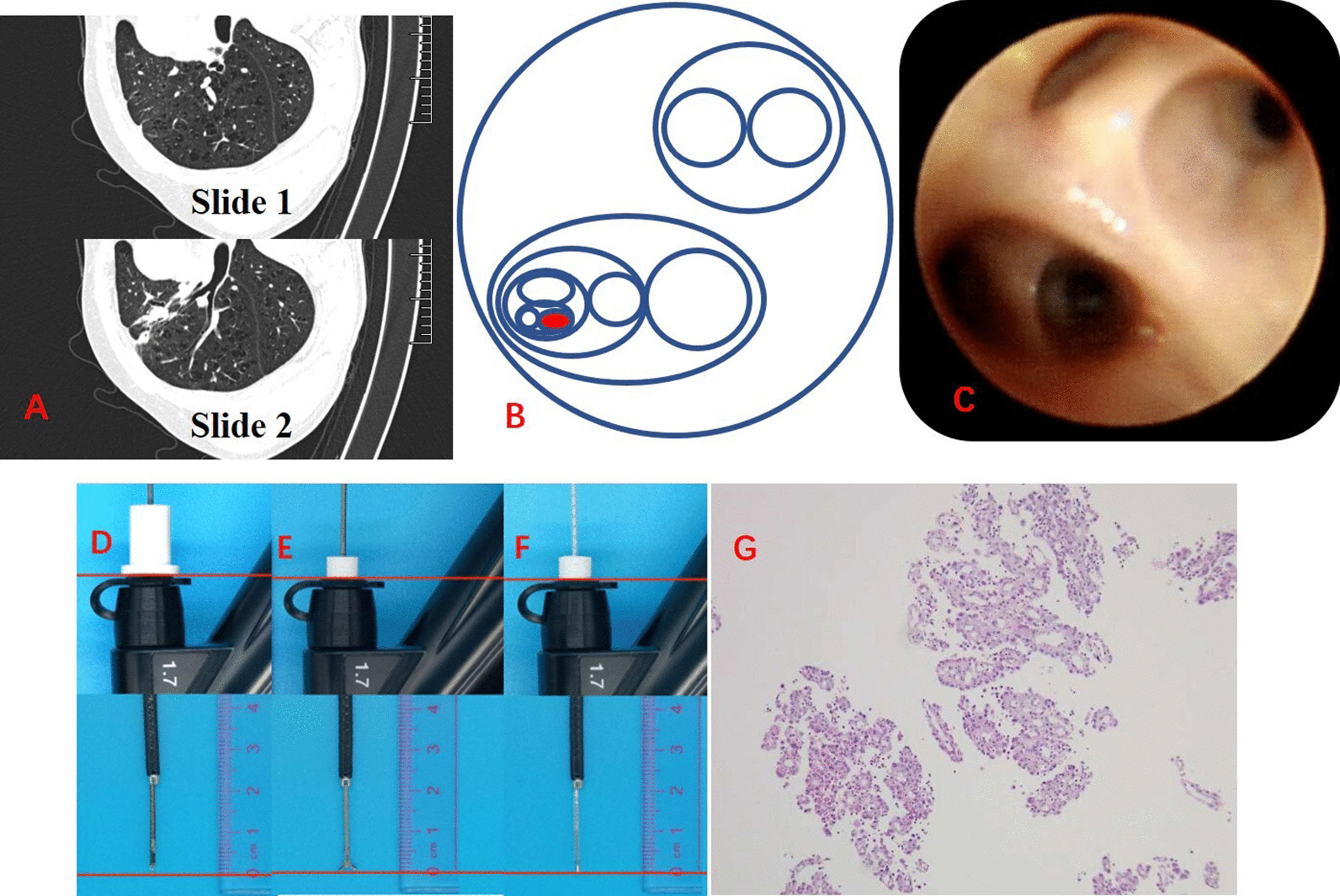
A case of patient with pulmonary nodule, the chest CT showed the lesion located in LB3bi. The chest CT slide was rotated by 90-degree clockwise (**A**), and the pathway to the target lung lesion was planned based upon the CT image (**B**). The opening of the left upper lobe assessed by bronchoscopy was in accordance with the rotated CT image (**A** and **C**). A radial ultrasound probe (**D**), biopsy forceps (**E**) and specimen brush (**F**) were respectively implemented for accurate positioning and tranbronchoscopic lung biopsy. The histopathological finding suggested pulmonary adenocarcinoma (**G**)

1. Hand-drawn bronchoscopic navigation planning
  1.1 CT image rotation and clock-scale numeralization
    As bronchoscopist standing at the patient’s head side, the chest CT slice (1-mm imaging slice thickness) should be rotated for the consistency of the spatial structure of CT image and bronchoscopic observation, as follows: (1) pulmonary lesion in the right upper lobe: 90-degree counter-clockwise rotation; (2) left upper lobe: 90-degree clockwise rotation; (3) lower lobe, right middle lobe and left lingual segment: 180-degree rotation; (4) no rotation in the dorsal segment. In CT images, the bronchial opening position is numerically marked based on the “clock” scale (Figs. 1 and 2).
  1.2 Hierarchical mapping for bronchoscopic navigation
    Each bronchial opening is graded and numerically marked according to CT images. The navigation mapping should be originated from the lobar bronchus which is drawn as a circle (the second generation of bronchus), subsequently, the next generation of bronchus is marked as a smaller circle located within the upper story (larger circle) and labelled by numeralization (Fig. 3B), and so forth, until reaching the target pulmonary lesion.
    The concrete operation instructions, in most instances, are as follows: (1) Right upper lobe: RB1 locates at the 12 o’clock position, RB2 at 3 o’clock and RB3 at 8 o’clock; (2) Right middle lobe: RB4 at 3 o’clock and RB5 at 9 o’clock; (3) Right lower lobe dorsal segment (RB6): rotate the bronchoscope to make the anterior subsegment of RB6 locate at the 9 o’clock position; (4) Right lower lobe: RB7 at 9 o’clock, RB8 at 12 o’clock, RB9 at 3 o’clock and RB10 at 6 o’clock; (5) Left superior lobe: LB1 at 10 o’clock, LB2 at 8 o’clock and LB3 at 3 o’clock; (6) Left lingual lobe: LB4 at 9 o’clock and LB5 at 3 o’clock; (7) Left lower lobe dorsal segment (LB6): rotate the bronchoscope to make the anterior subsegment of LB6 locate at the 3 o’clock position; (8) Lower left lobe: LB7 locates at the left of 12 o’clock position; LB8 at the right of 12 o’clock position; LB9 at 9 o’clock and LB10 at 6 o’clock (Figs. 1 and 2).
2. Bronchoscopy and navigation for sampling
  Bronchoscopy is performed under the guidance of navigation mapping, based on the hand-drawn bronchial segments with hierarchical numeralization. Once the bronchoscope reaches the target lesion or bronchus, a radial ultrasound probe with an outer diameter of 1.4 mm (UM-S20-17S, Olympus, Japan) would be advanced through the bronchoscopic working channel for accurate positioning and guiding tranbronchoscopic lung biopsy.
  A typical case who received HBN for guiding peripheral pulmonary nodule biopsy was shown in Fig. 3 and Additional file 1: Video 1.

### Virtual bronchoscopic navigation (VBN) procedure

The LungPoint Virtual Bronchoscopic Navigation System (Broncus Technologies, Inc., Mountain View, CA) was employed in this study. The HRCT images (1-mm slice thickness) were imported into the LungPoint system and automatically analyzed by the VBN software. The target lesion was marked on the CT image, which was segmented in three dimensions by the LungPoint software. Subsequently, the VBN system calculated up the potential pathways through the airway for approaching the target, guiding the bronchoscope advance to the pulmonary lesion under the direct observation [12].

### Comparison between VBN and HBN for guiding biopsy

Between March 2018 and October 2020, we retrospectively recruited patients with peripheral pulmonary nodules (larger than 8 mm in diameter) from The First Affiliated Hospital of Guangzhou Medical University. We excluded the subjects with ground-glass nodules, pulmonary nodules without bronchus sign or lesions were less than 1 cm away from the pleura. The enrolled subjects were divided into two groups in a chronological order (VBN and HBN group), mainly due to the ultrathin bronchoscope (BF-MP290F, Olympus, Tokyo, Japan) was available and implemented in clinical practice since Jun 2019 in our bronchoscopy center. Patients in VBN group were underwent bronchoscopy (P260F, Olympus, Tokyo, Japan) in conjunction with VBN and EBUS + guide sheath (GS) for navigation and positioning, while those in HBN group received ultrathin bronchoscopy and EBUS + GS under the guidance of HBN for biopsy. All the navigation procedures in this study were performed by one sophisticated bronchoscopist (Dr. Changhao Zhong). The demographic characteristics, procedural time, accessing performance and the diagnostic yield were analyzed and compared between VBN and HBN group.

This study was approved by the ethics committee of The First Affiliated Hospital of Guangzhou Medical University (Medical Ethics [Year 2017] No.81). All subjects gave written informed consent.

### Sample size

The sample size calculation was conducted using PASS 2021 software (UT, U.S.A). Using a non-inferiority margin of 10% and a one-sided alpha level of 5%, we calculated that at least 28 patients should be enrolled in each group (VBN and HBN group), which would provide the trial with 80% power to show the non-inferiority of the HBN method.

### Statistical analysis

Statistical analysis was performed using SPSS version 22.0 (IBM Corporation, Armonk, NY, USA) and GraphPad Prism 5.0 (GraphPad Inc., USA). Data were expressed as mean ± standard deviation or median (interquartile range) as appropriate. Comparison between two groups was analyzed by using *t*-test. Chi-squared and Fisher’s exact test were used for the analysis of categorical data as appropriate. *P* < 0.05 was deemed statistically significant unless otherwise stated.

## Results

### Demographic characteristics

Forty-eight patients with peripheral pulmonary nodule were enrolled in HBN group, while 42 in VBN group. The median age, gender, smoking status and cardiopulmonary comorbidities were comparable between HBN and VBN groups (all *P* > 0.05, Table 1).

**Table 1:** Demographic Characteristics

	VBN group	HBN group	*P*
Cases, No	42	48	
Age, median (IQR), y	59.62 (30–85)	56.75 (29–83)	0.53
Gender Male, No. (%)	23 (54.76%)	34 (70.83%)	0.11
Current smoker, No. (%)	25 (59.52%)	32 (66.67%)	0.48
Comorbidity			
Hypertension, No. (%)	17 (40.48%)	23 (47.92%)	0.48
Coronary artery disease, No. (%)	2 (4.76%)	4 (8.33%)	0.50
Congestive heart failure, No. (%)	3 (7.14%)	5 (10.42%)	0.59
COPD, No. (%)	8 (19.05%)	12 (25.00%)	0.50

*HBN* hand-drawn bronchoscopic navigation, *VBN* virtual bronchoscopic navigation, *COPD* chronic obstructive pulmonary disease

### Bronchoscopic navigation procedure

There were no significant differences between VBN and HBN group in terms of lesion size, location (generation of bronchi), radiographic type and distance from lesion to pleura (all *P* > 0.05, Table 2). Notably, the planning pathway time (1.32 *vs.* 9.79 min, *P* < 0.001) and total operation time (23.63 *vs.* 28.02 min, *P* = 0.002), as well as operating cost (758.31 ± 125.21 *vs.*1327.70 ± 116.25 USD, *P* < 0.001) were markedly less in HBN group, compared with those in VBN group.

**Table 2:** Comparison of bronchoscopic procedure between two groups

	VBN group	HBN group	*P*
Lobe^#^			
Right upper lobe, No. (%)	10 (23.81%)	10 (20.83%)	0.17
Right middle lobe, No. (%)	10 (23.81%)	4 (8.4%)	
Right upper lobe, No. (%)	10 (23.81%)	18 (37.50%)	
Left upper lobe, No. (%)	2 (4.76%)	6 (12.50%)	
Left upper lobe, No. (%)	10 (23.81%)	10 (20.83%)	
Location of lesion (IQR), generation*	6.60 (4–9)	6.44 (5–9)	0.50
Lesion size (IQR), mm*	22.19 (10.87–30.00)	20.19 (7.54–29.94)	0.15
Radiographic lesion type			
Solid	32 (76.19%)	33 (68.75%)	0.13
Subsolid	10 (23.81%)	15 (31.25%)	
RP-EBUS position			
Within the lesion	31 (73.81%)	35 (72.92%)	0.92
Adjacent to the lesion	11(26.19%)	13 (27.08%)	
Distance to pleura (IQR), cm*	10.50 (2.10–23.50)	8.70 (0.08–17.55)	0.28
Time of planning pathway, min*	9.79 (6.83–13.97)	1.32 (0.50–2.55)	**< 0.001**
Total operation time, min*	28.02 (20.92–41.48)	23.63 (5.48–24.55)	**0.002**
Operating cost (RMB)*	8229.44 ± 772.95	5041.97 ± 832.51	**< 0.001**

*Values are mean ± SD, or median (range)

Total operation time = Time of planning pathway + Bronchoscopic procedural time

Operating cost includes basic bronchoscopy, anesthesia, RP-EBUS, biopsy forceps with or without VBN

*HBN* hand-drawn bronchoscopic navigation, *VBN* virtual bronchoscopic navigation, *IQR* interquartile range, *RP-EBUS* radial probe endobronchial ultra sonography

### Diagnostic yield

**Fig. 4:**
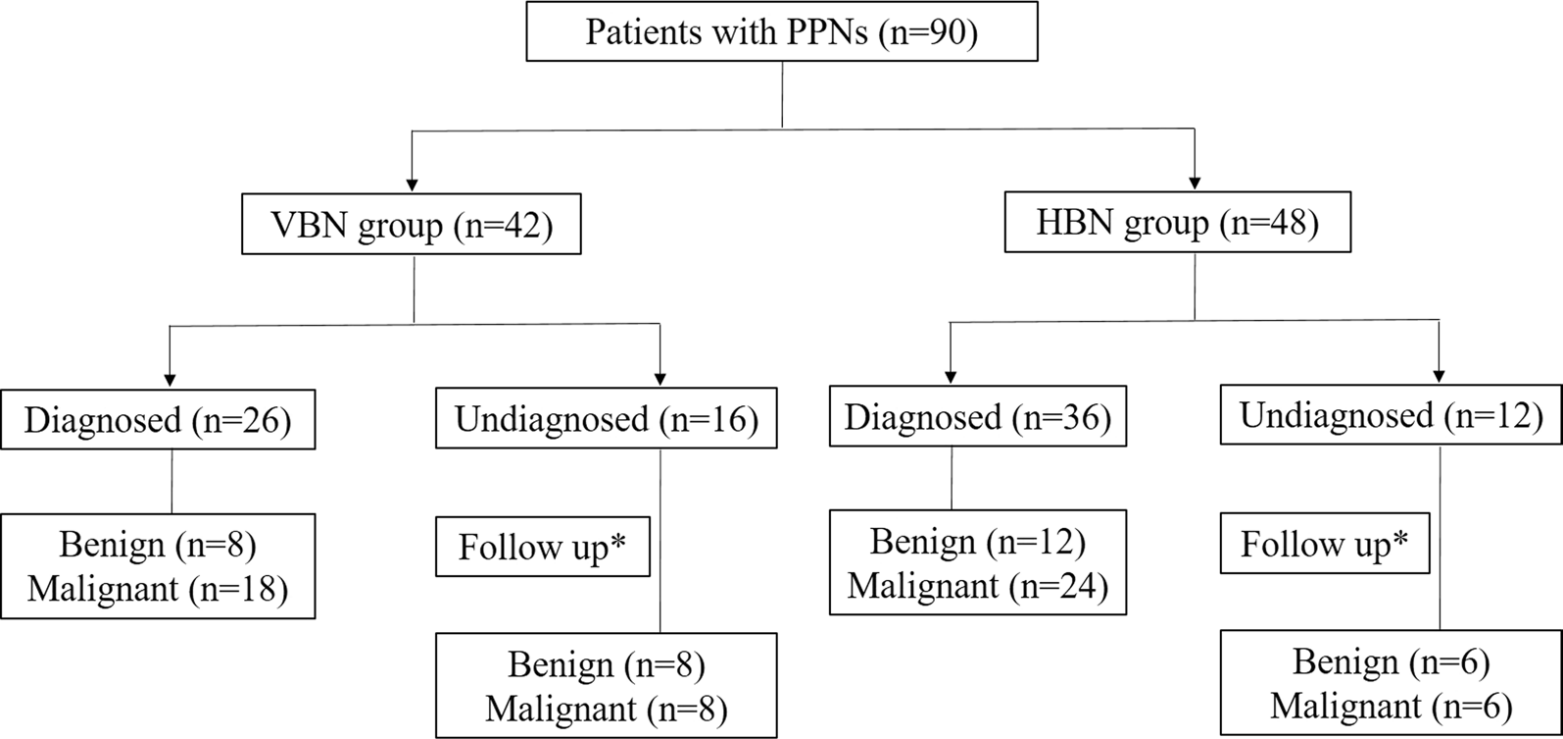
Flow chart of subject distribution and diagnosis. *Diagnosed by surgical resection or follow-up data. *PPN* peripheral pulmonary nodule, *HBN* hand-drawn bronchoscopic navigation, *VBN* virtual bronchoscopic navigation

The final diagnosis was confirmed by the pathologic findings obtained from bronchoscopic sampling, or relied on the subsequent surgery and follow-up data (Fig. 4). The pathological classification and etiology were compared between VBN and HBN group, but they did not achieve statistical significance (Table 3). The total diagnostic yield of HBN group had no marked difference from that of VBN group (75.00% *vs.* 61.90%, *P* = 0.25, Table 4). The pathological diagnostic efficiency of benign and malignant disease in HBN group appeared similar with those in VBN group, in irrespective of the size of pulmonary lesion (larger or smaller than 20 mm).

**Table 3:** Comparison of pathology and diagnostic efficacy between two groups

Pathology	Etiology		VBN group	HBN group
Diagnosed	Benign	Inflammation	5	3
		Tuberculosis	0	3
		Organizing pneumonia	0	1
		Aspergillus	0	1
		Granulomas	5	3
	**Malignant**	Adenocarcinoma	12	19
		Squamous	1	2
		small cell carcinoma	2	1
		Undifferentiated carcinoma	3	3
**Undiagnosed**	**Benign**	Inflammation	1	2
		Vasculitis	1	1
		Organizing pneumonia	2	0
		Tuberculosis	2	0
		Cryptococcus	0	1
		Pneumoconiosis	0	1
		Sarcoidosis	0	1
		Nocardia infection	0	1
	**Malignant**	Adenocarcinoma	3	1
		Squamous	0	2
		Undifferentiated carcinoma	5	2

*HBN* hand-drawn bronchoscopic navigation, *VBN* virtual bronchoscopic navigation

**Table 4:** Comparisons of lesion size, location and diagnostic yield

	VBN group	HBN group	*P*
Location, lobe			
Right upper lobe	5/10 (50.00%)	8/10 (80.00%)	0.35
Right middle lobe	8/10 (80.00%)	3/4 (75.00%)	0.56
Right upper lobe	7/10 (70.00%)	12/18 (66.67%)	1.00
Left upper lobe	2/2 (100.00%)	5/6 (83.33%)	1.00
Left upper lobe	6/10 (60.00%)	7/10 (70.00%)	1.00
Radiographic lesion type			
Solid	20/32 (62.50%)	23/33 (69.70%)	0.61
Subsolid	6/10 (60.00%)	12/15 (80.00%)	0.28
Benign lesions			
< 20 mm	4/8 (50.00%)	6/11 (54.55%)	> 0.99
> 20 mm to < 30 mm	4/8 (50.00%)	6/7 (85.71%)	0.14
Total	8/16 (50.00%)	12/18 (66.67%)	0.49
Malignant lesions			
< 20 mm	5/8 (62.50%)	11/14 (78.57%)	0.62
> 20 mm to < 30 mm	13/18 (72.22%)	13/16 (81.25%)	0.69
Total	18/26 (69.23%)	24/30 (80.00%)	0.35
All lesions			
< 20 mm	9/16 (56.25%)	17/25 (68.00%)	0.52
> 20 mm to < 30 mm	17/26 (65.38%)	19/23 (82.61%)	0.21
Total	26/42 (61.90%)	36/48 (75.00%)	0.25

Data are shown as numbers of lesions with confirmed diagnosis/total lesions (%)

*HBN* hand-drawn bronchoscopic navigation, *VBN* virtual bronchoscopic navigation

### Complications

In HBN group, one case of pneumothorax, not requiring chest drainage, was identified by chest X-ray examination after transbronchial lung biopsy. No other complications (hemorrhage, procedure-related pneumonia or arrhythmia, etc.) were reported during or after the bronchoscopic navigation procedure.

## Discussion

In this study, we have established a novel hierarchical clock-scale hand-drawn mapping method for bronchoscopic navigation, which could emerge as a simple, economical and feasible guiding modality for pulmonary nodule biopsy. The navigation mapping with ultra-thin bronchoscopy provided a more distal airway detection and a diagnostic yield of 75% for peripheral pulmonary lesion.

Peripheral pulmonary nodule, with an increasing prevalence, presents a particular challenge for tissue biopsy and accurate diagnosis. Bronchoscopic navigation systems (VBN, ENB, etc.) were recently implemented to guide transbronchial lung biopsy with a good diagnostic yield, but requiring high consumption and long training time [13, 14], which might limit the widely use of navigation method for lung biopsy in majority of general hospitals. A novel navigation modality with simple and fast preparation is currently required further exploration.

In the previous study, Prof. Noriaki Kurimoto had first introduced the bronchial branch tracing method for bronchoscopic diagnosis [11]. Subsequently, Zhang et al*.* reported a manual mapping method for guiding pulmonary nodule biopsy in the clinical practice [15]. Whereas, the manual mapping might be mainly based upon individual experience, lacking a standard operating procedure (SOP) and quantitative guideline for accurate navigation. Hence, in review of previously published research, we aimed to establish a normative approach of hierarchical clock-scale hand-drawn mapping for bronchoscopic navigation based on the “clock” scale, making it feasible for accurately guiding pulmonary lesions along with bronchial generations. Since bronchoscopists have various operation habits and standing positions, the SOP of navigation mapping would provide a standardized approach for guiding transbronchial lung biopsy.

The VBN system might have limitation in planning a pathway to the peripheral airways (generally less than 3 mm in diameter), which is associated with the finite resolution of CT imaging for detecting the sixth generation bronchi or more distal airways [16, 17]. Furthermore, CT measurement algorithm errors, airway structural variation and sputum blocking could commonly give rise to the deviation of planned path in navigation system. By contrast, the navigation mapping method is conducted on the basis of imaging reading by sophisticated operator, which could reduce the magnitude of limitation by CT image artifacts and airway luminal secretion. Taking this into account, it is therefore plausible that the bronchoscopic navigation mapping could provide an deep detection of the distal airways. Moreover, compared to VBN system, the navigation mapping method occupied markedly less time for planning a pathway along with bronchial generations and guiding to the target pulmonary lesion, making it feasible and simple to perform the bronchoscopic navigation with time-saving and easy preparation.

The preliminary clinical use of navigation system (VBN, ENB, etc.) with routine bronchoscope might confer a limitation for detecting peripheral pulmonary nodule in the more distal airways. Until fairly recently, the introduction of ultra-thin bronchoscope, with outside diameter of 3.0 mm, was reportedly conducive to access peripheral airways, expanding the bronchial inspection and improving the diagnostic yield of peripheral pulmonary lesions [7, 18, 19]. In the current study, HBN in conjunction with ultra-thin bronchoscope, accessing to the 7th generation or more distal airways, provided a diagnostic yield of 75% for peripheral pulmonary nodule, which was compatible with that of routine bronchoscopy with virtual navigation. This lent support to the possibility that HBN combined with ultra-thin bronchoscope could serve as a potential surrogate for virtual bronchoscopic navigation system in the majority of general hospitals with cost–benefit consideration.

Some caveats should be considered. The current study was conducted in a single medical center with small sample sizes, which might limit the generalizability of our findings. Secondly, we performed comparisons between VBN + routine bronchoscope and HBN + ultrathin bronchoscope, which was recently implemented in clinical practice, rather than using ultrathin bronchoscope in both groups, might have slightly biased the results. Whereas, it would not weaken the tenability of the conclusion that HBN provides an analogous value for guiding peripheral pulmonary nodule biopsy compared with VBN system.

## Conclusion

Hierarchical clock-scale hand-drawn mapping for bronchoscopic navigation serves as a feasible and economical method for guiding peripheral pulmonary nodule biopsy, it could be conducive to access more distal airways and achieve similar diagnostic yield in comparison with virtual bronchoscopic navigation.

## Supplementary Information

**Additional file 1. Video 1.** A typical case who received HBN for guiding peripheral pulmonary nodule biopsy.

## Data Availability

The datasets used and analyzed in the current study are available from the corresponding author on reasonable request.

## Abbreviations

VBN: Virtual bronchoscopic navigation
ENB: Electromagnetic navigation bronchoscopy
RP-EBUS: Radial probe endobronchial ultrasound
HBN: Hand-drawn bronchoscopic navigation
HRCT: High-resolution computer tomography
GS: Guide sheath
SOP: Standard operating procedure
